# Self-management support interventions for patients with cancer treatment-related oral mucositis: a scoping review

**DOI:** 10.3389/fonc.2026.1950737

**Published:** 2026-10-02

**Authors:** Han Yan, Xu Liu, Jiaying Li, Dongchi Ma, Wenjie Wang, Yuxing Xie, Li Wang, Li Ning

**Affiliations:** 1 School of Nursing, Zhejiang Chinese Medical University, Hangzhou, Zhejiang, China; 2 Nursing Department, Hangzhou First People’s Hospital, Hangzhou, Zhejiang, China

**Keywords:** cancer, oral mucositis, scoping review, self-management, supportive care

## Abstract

**Objective:**

Cancer treatment-related oral mucositis (OM) is one of the most common adverse effects of cancer treatment and can substantially impair patients’ quality of life. Its effective management depends heavily on patients’ continuous self-management. This scoping review aimed to systematically map the intervention components, delivery modes, and existing limitations of self-management support interventions, thereby providing evidence to inform the optimization of intervention strategies.

**Methods:**

A scoping review was conducted in accordance with the PRISMA Extension for Scoping Reviews. Seven databases, including PubMed, Cochrane Library, Web of Science, CINAHL, Embase, China National Knowledge Infrastructure, and WanFang, were searched from inception to May 10, 2026. Two reviewers independently screened titles, abstracts, and full texts according to predefined inclusion and exclusion criteria. Data were charted using a standardized form and summarized using descriptive numerical and content analyses.

**Results:**

A total of 7,132 records were identified, and 16 studies were ultimately included, comprising 9 quasi-experimental studies, 6 randomized controlled trials, and 1 mixed-methods study. The interventions were primarily hospital-based and nurse-led, with some incorporating digital tools or written materials. Reported outcomes generally included OM-related clinical outcomes, quality of life, self-management-related outcomes, and nutritional status, although the direction and magnitude of findings varied across studies.

**Conclusions:**

Existing studies have reported potential improvements in OM-related and self-management-related outcomes following self-management support interventions. However, these findings should be interpreted cautiously because of heterogeneity in study designs, intervention components, and outcome measures, together with the absence of critical appraisal. Further well-designed studies using standardized outcome measures are needed to strengthen the evidence base.

## 1 Introduction

Cancer treatment-related oral mucositis (OM) is among the most common complications in patients with cancer undergoing radiotherapy, anticancer drug therapy, or hematopoietic stem cell transplantation (1). Currently, OM occurs in nearly all patients receiving radiotherapy and in 24.8–67.0% of those receiving chemotherapy (2), with the incidence reaching 90–100% among patients with head and neck tumors undergoing radiotherapy (3). Its main manifestations include erythema, erosion, ulceration, and pain of the oral mucosa (4), In severe cases, OM can lead to eating difficulties, nutritional impairment, and even interruption of anticancer treatment, thereby substantially compromising patients’ quality of life and treatment outcomes (5). With the development of supportive oncology care, the management of OM has gradually shifted from symptomatic treatment alone to early prevention and comprehensive management (6). Because the onset and progression of OM are dynamic processes, its management involves multiple aspects, including symptom monitoring, pain management, and daily self-care.Therefore, patients’ self-management ability plays an important role in disease management (7). Self-management refers to the ability of individuals to manage symptoms, treatment, physiological and psychosocial changes, and lifestyle adjustments as they cope with the progression of a chronic condition (8). Recent studies have shown that OM can be effectively prevented and managed through self-management support interventions (9). However, although intervention studies on self-management of OM among patients with cancer have gradually increased, substantial differences remain in intervention components, delivery modes, and outcome measures. Therefore, this study aimed to systematically synthesize the existing evidence in this field, identify current limitations and research gaps, and provide directions for optimizing future intervention programs and research designs, with the ultimate goal of improving patients’ self-management ability, symptom outcomes, and quality of life.

## 2 Methods

### 2.1 Research design

This scoping review was guided by the five-stage framework proposed by Arksey and O’Malley and the current JBI methodological guidance for scoping reviews (10, 11). The review process included identifying the research question; searching for and identifying relevant studies; selecting relevant studies; charting the data; and collating, summarizing, and reporting the results. Our primary objective was to comprehensively map the existing literature and systematically summarize the intervention components, delivery modes, and limitations of self-management support interventions for OM, thereby providing a foundation for further optimizing intervention strategies. The results of the review were reported following the Preferred Reporting Items for Systematic Reviews and Meta-Analyses of scoping review guidelines (11).

The review protocol was prospectively registered with the Open Science Framework (OSF) before study selection and data charting and is available at https://doi.org/10.17605/OSF.IO/K3ZY5.

As no original data were collected from human or animal subjects, ethical approval was not required for this work.

### 2.2 Research question

Based on the scoping review title and identified objectives, a scoping review was conducted to answer the research questions:

1. What self-management support interventions are available for patients with OM? What are their characteristics (e.g., intervention components, delivery mode, implementer, setting, frequency, and duration)?
2. What outcome measures and assessment tools have been reported, and what outcomes have individual studies reported following these interventions?
3. What are the current limitations and research gaps in this field?

### 2.3 Inclusion and exclusion criteria

Eligibility criteria were structured according to the Population/Participants–Concept–Context (PCC) framework recommended by JBI.

The inclusion criteria for this review were as follows:

1. Participants: The study population consists of patients undergoing cancer treatment who have developed or are at risk of developing OM, with no age restrictions;
2. Concept: Self-management support was defined as interventions that enable patients to actively prevent or manage OM through self-monitoring, self-care, or other active management behaviors. Patient education alone, or clinician-led care without explicit patient participation, was not considered sufficient for inclusion.
3. Context: Study settings include inpatient, outpatient, community, or home care environments;
4. Types of studies: Original studies such as randomized controlled trials, quasi-experimental studies, and mixed-methods studies were included.

The exclusion criteria were as follows:

1. Duplicate publications;
2. Publications for which the full text is unavailable;
3. Publications in which the raw data are unclear, incomplete, or from which key study results cannot be extracted;
4. Interventions limited to didactic patient education or predominantly clinician-led care without active patient participation in self-management were excluded.

### 2.4 Search strategy

The research team conducted a systematic search of seven databases—PubMed, the Cochrane Library, Web of Science, CINAHL, Embase, China National Knowledge Infrastructure (CNKI), and Wanfang—covering the period from the inception of each database to May 10, 2026. To ensure the inclusion of a broad range of relevant studies, no language or publication date re­strictions were applied during the search process. A combination of Medical Subject Headings (MeSH)/Emtree terms and free-text terms was utilized to construct the search queries. For a detailed description of the search strategies specific to each database, please refer to the **Supplementary Material** (**Supplementary File 1**).

### 2.5 Study selection and data charting

All retrieved records were imported into EndNote 20 for management and deduplication. Two reviewers (HY and XL) independently screened titles and abstracts and subsequently assessed full texts against the predefined eligibility criteria. Disagreements were first resolved through discussion; if consensus could not be reached, a third reviewer (DM) was consulted.

A standardized data-charting form was developed by HY and XL based on the review questions and predefined data items and was pilot-tested by both reviewers on a small sample of included studies before formal charting. The form was refined as necessary following the pilot test. Two reviewers independently charted study characteristics, including author, country or region, publication year, study design, sample size, intervention characteristics, intervention duration and frequency, and outcome measures. Discrepancies in data charting were resolved through discussion. Chinese-language records were screened and assessed in the original Chinese by HY and XL, and data from eligible Chinese-language studies were charted directly from the original-language full texts rather than from translated versions. The same eligibility criteria and data-charting procedures were applied to both Chinese- and English-language studies.

### 2.6 Data synthesis

The charted data were summarized using descriptive numerical analysis and descriptive content analysis. Study characteristics were summarized using frequencies. Intervention categories were developed inductively from the charted intervention descriptions according to the predominant delivery mode. Multicomponent interventions were classified according to their primary delivery mode, and disagreements in categorization were resolved through discussion.

## 3 Results

### 3.1 Literature search and selection process

The database searches identified 7,132 records. After removal of 983 duplicates, 6,149 records underwent title and abstract screening, of which 6,041 were excluded because they were not relevant to the review topic (n = 5,606) or were of an ineligible publication type (n = 435). A total of 108 reports were sought for retrieval. Of these, 32 reports could not be retrieved, including 14 for which the full text could not be located or accessed and 18 for which only an abstract was available. The remaining 76 reports were assessed for eligibility. Following full-text assessment, 60 reports were excluded because of an ineligible population (n = 35), an ineligible intervention (n = 21), or incomplete full-text information that precluded eligibility assessment (n = 4). Ultimately, 16 studies were included in the review (**Figure 1**).

**Figure 1:**
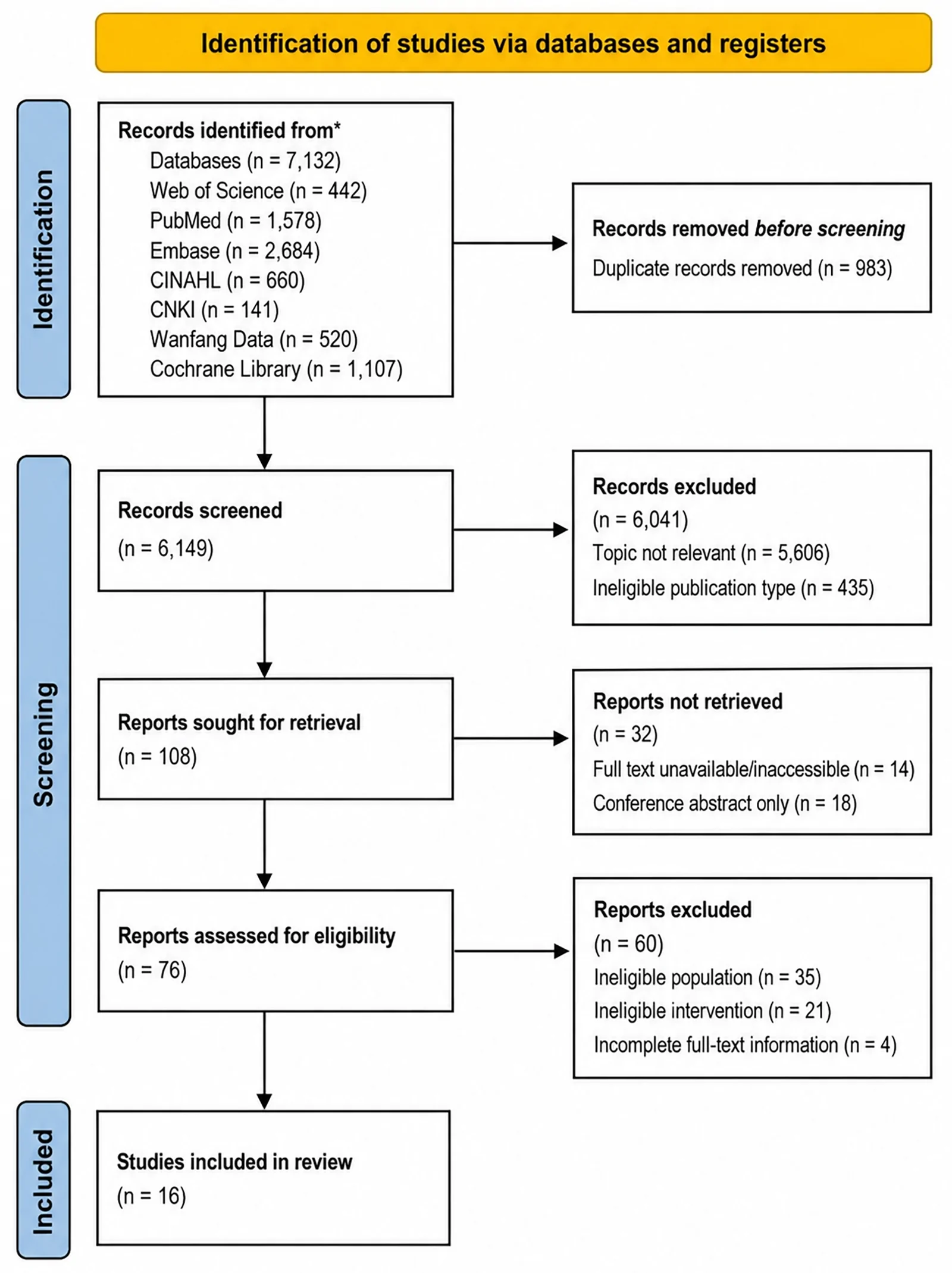
Flow diagram illustrating the study-selection process of screening and identification of studies.

### 3.2 Characteristics of sources of evidence

The included studies were published between 2016 and 2025, showing an overall increasing trend in recent years. Most studies were conducted in China, including nine from mainland China and one from Taiwan, China; the remaining studies were conducted in Turkey (n = 2), Germany (n = 2), the United States (n = 1), and Brazil (n = 1). Regarding study design, the included studies were predominantly quantitative, comprising quasi-experimental studies (n = 9) and randomized controlled trials (n = 6), along with one mixed-methods study (n = 1). **Table 1** summarizes the basic characteristics, intervention details, and outcome measures of the included studies.

**Table 1:** Summary of interventions for patients with cancer treatment-related oral mucositis.

Author and year	Study design	Country or region	Sample size	Intervention	Intervention duration	Outcome measures/tools
Kara (12), 2023	Quasi-experimental	Turkey	27/—	Radiotherapy nurse-led oral mucositis management: pre-radiotherapy oral examination and dental assessment; provision and explanation of the Guide to the Prevention and Care of Oral Mucositis; approximately 30-minute weekly oral care training from the start of radiotherapy, with individualized consultation and follow-up; management plans dynamically adjusted according to patients’ problems.	6 weeks	WHO Oral Toxicity Scale; Oral Mucositis Follow-up Form; Nutritional Risk Screening (NRS); body weight; stress rating; treatment-compliance assessment.
Leppla (13), 2016	Randomized controlled trial	Germany	9/9	Two educational and behavioral intervention sessions were delivered 1 week before admission and on the day of admission. The intervention included written educational materials on oral mucositis, instruction on the use of the oral mucositis questionnaire, guidance on daily self-assessment of oral status during hospitalization, and selection of corresponding oral care strategies according to assessment results. On the day of admission, patients were instructed in the modified Bass toothbrushing technique, self-management goals were jointly established, and mobile phone reminders were used when adherence declined.	28 days	WHO oral mucositis scale; adapted Basel Assessment of Adherence Scale (BAASIS); visual analogue scale (VAS) for adherence; modified Quigley-Hein Plaque Index (QPI).
Yüce (14), 2019	Randomized controlled trial	Turkey	30/30	Oral mucositis prevention education based on a patient education booklet, including nutritional advice; management of xerostomia and alterations in taste and smell; toothbrushing and flossing techniques; preparation of mouth rinse; and daily self-assessment of oral mucosa. Ongoing guidance was provided by telephone or face-to-face during follow-up.	From day 1 of chemotherapy to day 21	Oral Assessment Guide (OAG); EORTC QLQ-C30 version 3.0.
Lin (15), 2022	Quasi-experimental	China	32/32	A mobile oral care application was used for 2 months in addition to routine care. The application comprised 4 modules: (1) health education (etiology and impact, assessment tools, Bass toothbrushing video, pain care recommendations, rehabilitation exercise videos); (2) self-recording (World Health Organization oral mucositis grading, numeric rating scale for pain, Patient-Generated Subjective Global Assessment, etc); (3) graded oral care, providing individualized recommendations according to mucositis grade; and (4) a discussion platform for symptom trend visualization and communication with nurses via the LINE application.	2 months	Oral mucositis grading; Numeric Rating Scale (NRS) for pain; Patient-Generated Subjective Global Assessment (PG-SGA); body weight/weight loss; hemoglobin; albumin; quality-of-life questionnaire.
Schmidt (16), 2016	Quasi-experimental	Germany	42/37	An interdisciplinary self-management intervention for hematopoietic stem cell transplantation was implemented in addition to routine care. This included written materials on oral care, daily self-assessment of oral status using an oral mucositis questionnaire, and completion of oral care procedures for mucositis prevention. Nutritional support was also included: patients monitored changes in appetite and nutrition, while nurses provided guidance in response to patients’ questions or needs.	During hospitalization	EORTC QLQ-C30 version 3.0; CTCAE version 3.0 for oral mucositis; Oral Mucositis Daily Questionnaire (OMDQ, intervention group); BMI and laboratory nutritional indicators; Physical Activity Questionnaire/activity log; Fatigue Symptom Inventory (FSI); patient-satisfaction NRS; length of hospital stay.
Ruegg (17), 2021	Mixed-methods study	United States	11/—	Symptom management education was first provided, followed by a nurse-led individualized telephone intervention with twice-weekly phone guidance to alleviate oral mucositis and prevent dehydration.	From the treatment period to 4 weeks after treatment completion	MD Anderson Symptom Inventory—Lung Cancer/Head & Neck Cancer (MDASI-LC/MDASI-HN); Functional Assessment of Cancer Therapy—Lung/Head & Neck (FACT-L/FACT-H&N); adapted Chronic Disease Self-Efficacy Scale; Partners in Health Scale; unscheduled medical visits.
Yao Dan (18), 2018	Randomized controlled trial	China	41/41	The intervention group received the Orem nursing model. Appropriate nursing systems were provided according to activities of daily living/Barthel scores, including pre-radiotherapy health education, oral care and functional exercise guidance during radiotherapy, and post-radiotherapy oral self-examination and assessment.	Throughout the course of radiotherapy	Oral mucositis incidence/severity grading; self-care ability scale/questionnaire (specific tool not reported in the available article information).
Guo Ningning (19), 2023	Quasi-experimental	China	53/53	Orem self-care theory-based intervention was implemented in addition to routine intervention, including wholly compensatory, partly compensatory, and support-educative systems, combined with individualized oral care, dietary guidance, symptom observation, emotional support, and health education.	During radiotherapy and chemotherapy	Self-care ability scale/questionnaire; oral mucositis incidence/severity grading; quality-of-life scale (specific tool names not clearly reported in the available article information).
Shen Hui (20), 2016	Randomized controlled trial	China	50/50	Self-care intervention based on Orem’s self-care theory was implemented in addition to routine oral care. According to patients’ self-care ability, wholly compensatory, partly compensatory, and support-educative systems were adopted to provide oral care guidance, fluid and dietary guidance, symptom observation, and psychological support.	During radiotherapy and chemotherapy	Self-care ability assessment (self-care skills, responsibility, self-concept, and health-knowledge dimensions); oral mucositis grading; visual analogue scale (VAS) for oral pain.
Bezerra (21), 2021	Quasi-experimental	Brazil	14/14	The Oral Health Education and Prevention Program was implemented, including verbal education, games, video demonstrations, positive reinforcement, and weekly oral assessments, to promote oral hygiene behaviors and prevent oral mucositis in pediatric oncology patients.	10 weeks	Modified Oral Assessment Guide (OAG).
Wang Huan (22), 2018	Randomized controlled trial	China	42/42	The teach-back method was used for oral mucositis prevention education. A preventive care team repeatedly explained the content, asked patients to repeat it back, provided corrective feedback, and supplemented the intervention with an educational ruler for oral mucositis assessment.	During hospitalization	Investigator-developed knowledge/behavior assessment; oral mucositis assessment/grading ruler.
Wang Qiaorong (23), 2025	Quasi-experimental	China	90/90	Continuity of care based on the Omaha System was implemented in addition to routine care, including establishment of personal records, WeChat group management, phased health education, guidance on oral mucositis care, dietary and medication guidance, video-based education, and continuous follow-up.	2 cycles (60 days)	Self-management ability scale; oral mucositis incidence/severity assessment; onset/duration records (specific instrument names not clearly reported in the available article information).
Zhao Shunzhen (24), 2019	Quasi-experimental	China	35/36	An intervention combining health education prescriptions with patient record booklets was implemented on the basis of routine care and health education. Educational prescriptions were issued according to radiotherapy stage, and record booklets were used to supervise mouth rinsing, oral observation, and documentation of dietary changes.	Throughout the course of radiotherapy	NCI Common Terminology Criteria for Adverse Events (NCI-CTCAE) version 4.0 for oral mucositis; mouth-rinsing execution/compliance rate recorded in the patient record booklet.
Deng Saijiao (25), 2022	Quasi-experimental	China	51/51	An intervention combining health education prescriptions with a mobile diary was applied in addition to routine health education and radiotherapy nursing, including oral assessment, phased distribution of educational prescriptions, toothbrushing and mouth-rinsing guidance, oral functional training, and daily recording of intervention adherence.	3 radiotherapy cycles	Oral mucositis grading; nutrition-related indicators; self-care/adherence records from the mobile diary (specific scale names not clearly reported).
Xiao Wenzhi (26), 2017	Quasi-experimental	China	35/35	A ward-based health education television channel was established on the basis of routine health education, repeatedly broadcasting PowerPoint presentations and short videos on radiotherapy precautions, oral functional exercises, demonstrations of toothbrushing and mouth rinsing, dietary guidance, and oral self-assessment and self-examination.	During radiotherapy	Investigator-developed disease-knowledge/behavior assessment; oral mucositis grading/assessment.
Chen Zimin (27), 2021	Randomized controlled trial	China	50/50	The intervention group received early health education and nursing intervention, whereas the control group received routine care and health education. The intervention focused on early oral care education, promotion of adherence, and self-management guidance.	1 week before radiotherapy	Oral mucosal reaction grading; oral-care correct-execution rate; self-management behavior assessment.

Sample size is presented as intervention/control; — indicates not applicable or no control group reported. BAASIS, Basel Assessment of Adherence Scale; BMI, body mass index; CTCAE, Common Terminology Criteria for Adverse Events; EORTC QLQ-C30, European Organisation for Research and Treatment of Cancer Quality of Life Questionnaire Core 30; FACT, Functional Assessment of Cancer Therapy; FSI, Fatigue Symptom Inventory; MDASI, MD Anderson Symptom Inventory; NCI, National Cancer Institute; NRS, Numeric/Nutritional Risk Screening as specified; OAG, Oral Assessment Guide; OMDQ, Oral Mucositis Daily Questionnaire; PG-SGA, Patient-Generated Subjective Global Assessment; QPI, Quigley-Hein Plaque Index; VAS, Visual Analogue Scale; WHO, World Health Organization.

### 3.3 Characteristics of the interventions

#### 3.3.1 The theoretical foundation of the interventions

The included studies showed substantial heterogeneity in the theoretical foundations underpinning self-management support interventions. Three studies implemented interventions guided by Orem’s self-care theory, with a focus on the wholly compensatory system, partly compensatory system, and supportive-educative system (18–20). Another study was based on the continuum of care within the Omaha System, using it as an overarching framework for problem classification, intervention delivery, and phased management (23). Most studies, however, did not report a clear theoretical foundation (n = 12) (12, 14–17, 21, 22, 24–27).

#### 3.3.2 Intervention setting

Since many patients are undergoing radiation therapy, chemotherapy, or transplantation, interventions are often conducted in tandem with hospital treatment protocols. Existing interventions are primarily implemented in hospitals or treatment-related settings, including radiation oncology departments, chemotherapy wards, hematopoietic stem cell transplantation units, and general inpatient wards (12–14, 16, 18–22, 24–27). Some studies have extended interventions from inpatient settings to outpatient or home-based environments by using telephone calls, WeChat, and mobile applications to provide ongoing support and follow-up (14, 15, 17, 22, 23, 25). Overall, these interventions remain predominantly hospital-based; however, with the increasing use of digital technologies, their scope is gradually expanding to include home care and post-discharge rehabilitation follow-up.

#### 3.3.3 Intervention delivery characteristics and components

The included self-management support interventions varied in their components and delivery characteristics, including nurse-led interventions, digital tool-assisted interventions, written material-supported interventions, and multidisciplinary collaborative interventions. Eight studies provided comprehensive nurse-led self-management support, including oral care instruction, symptom monitoring, dietary management, and medication management (12, 14, 17–20, 22, 27). Five studies used digital tools to support interventions, mainly through mobile applications, WeChat, and educational videos to provide health education and behavioral supervision (15, 21, 23, 25, 26). Two studies used written prescriptions or recording tools, in which written educational materials and patient record booklets were provided to guide patients in oral self-management (13, 24). In addition, one study adopted a multidisciplinary collaborative intervention delivered by exercise therapists and nurses; exercise therapists developed exercise and relaxation programs for patients, while nurses provided oral management guidance and nutritional support (16).

#### 3.3.4 Intervention duration and frequency

Regarding intervention timing and duration, the interventions included in this review typically began 1 week before treatment, on the day treatment was initiated, or during hospitalization, with durations ranging from 1 day to 3 months. Among these, two studies implemented interventions between 1 week before admission and the day of admission (13, 27). In six studies, interventions began at admission or at the start of treatment and continued for 1–3 months after treatment (12, 14, 15, 17, 21, 23). The remaining eight studies primarily implemented interventions during hospitalization or during radiotherapy and chemotherapy (16, 18–20, 22, 24–26). Regarding intervention frequency, the included studies ranged from a single educational session to continuous reinforcement, including two educational sessions (13), twice-weekly telephone follow-ups (17), weekly assessments or training sessions (12, 21), and health education broadcasts delivered three times per week (26). However, six studies did not explicitly report the duration of each intervention session or the specific frequency of implementation, describing the interventions only in general terms as being delivered continuously throughout the treatment period (16, 18–20, 24, 25). Overall, considerable heterogeneity was observed in the timing, duration, and frequency of self-management support interventions for OM.

#### 3.3.5 Outcome measures and reported findings

The outcome measures reported in the included studies were categorized into four domains: oral mucositis-related clinical outcomes, quality of life, self-management-related outcomes, and nutritional status. All 16 studies reported oral mucositis-related clinical outcomes, mainly including the incidence, severity, onset, and duration of oral mucositis (12–27). Commonly used assessment tools included the World Health Organization oral mucositis grading scale, the Oral Assessment Guide (OAG), the Common Terminology Criteria for Adverse Events version 3.0 (CTCAE v3.0), and the Radiation Therapy Oncology Group (RTOG) acute radiation morbidity scoring criteria. Four studies reported quality-of-life outcomes, mainly using the EORTC QLQ-C30, EORTC QLQ-H&N35, and QOL-CSV (14–16, 19). Thirteen studies reported self-management-related outcomes, including self-management ability, knowledge, self-efficacy, and behavioral adherence (12, 13, 16–20, 22–27). Commonly used tools included the Exercise of Self-Care Agency Scale, the Adult Health Self-Management Ability Scale, the General Self-Efficacy Scale, and investigator-developed questionnaires. Nutritional status outcomes were reported in 4 studies and were mainly assessed using the Nutritional Risk Screening 2002, PG-SGA, BMI, and biochemical indicators such as hemoglobin, albumin, and prealbumin (12, 15, 16, 25).

The reported findings varied across individual studies and outcome domains. Several studies reported favorable changes following the interventions. For example, Yüce reported a lower frequency of oral mucositis in the intervention group, together with better functional and general health status and lower symptom scores than in the control group (14). Lin et al. reported less severe oral mucositis, lower pain scores at later assessments, and more favorable nutritional indicators in patients receiving the mobile-app intervention (15). However, the findings were not uniformly favorable. Schmidt et al. reported no between-group difference in the primary outcome of health-related quality of life at discharge (16), although non-confirmatory *post hoc* analyses suggested shorter hospitalization and greater activity in the intervention group. Leppla et al. reported only small effect sizes for the highest oral mucositis grade, duration, and onset, while adherence declined over time in both groups (13). Bezerra et al. also reported intervention-related differences in oral mucositis outcomes, although the findings concerning severity were not uniformly favorable (21). Thus, the available evidence included favorable, null, and mixed findings, and the results are presented here as outcomes reported by individual studies rather than as evidence of established overall intervention effectiveness.

### 3.4 Comparative synthesis of self-management functions and clinical contexts

The 16 included studies supported different aspects of self-management. Education and oral self-care skills were the most common components across interventions (12–27), while several interventions additionally incorporated self-monitoring or self-assessment, individualized guidance, adherence support, and ongoing follow-up (13, 15–17). Explicit self-monitoring was incorporated through approaches such as daily oral assessment, symptom recording, oral self-examination, or treatment diaries (13–16, 18, 24–26). Tailoring was evident in interventions that adjusted management according to mucositis severity, self-care ability, treatment stage, or individual problems (12, 15, 18–20, 24). In contrast, explicit goal setting and action planning were less frequently reported, with these elements being most clearly described in the OrCaSS intervention (13). Considerable variation was also observed across populations and treatment contexts. Ten studies involved patients receiving radiotherapy or chemoradiotherapy, predominantly for head and neck or nasopharyngeal cancer (12, 15, 17–20, 24–27); three involved hematopoietic stem cell transplantation (13, 16, 23); two focused on chemotherapy (14, 22); and one specifically involved pediatric patients (21). Most interventions were delivered during hospital-based treatment, whereas only a limited number explicitly extended support beyond the treatment setting through telephone follow-up, mobile applications, or WeChat-based continuity of care (15, 17, 23, 25).

The goals of the interventions also differed. Most focused primarily on prevention through oral hygiene, dietary guidance, symptom observation, and adherence to preventive care (14, 18, 21, 22, 24–26), whereas fewer incorporated responsive symptom-management strategies based on patients’ changing symptoms or assessment results (12, 15, 17). Caregiver involvement was not explicitly reported as a structured intervention component, and health literacy or digital access was not formally assessed or used to tailor interventions across the included studies.

## 4 Discussion

This scoping review mapped 16 studies on self-management support for OM and identified substantial variation in the functions supported, patient populations, treatment contexts, delivery modes, and outcome measurement, and outcome measurement. Overall, existing interventions predominantly emphasized education, oral self-care, symptom monitoring, and behavioral support, whereas more comprehensive self-management functions and cross-setting continuity were less consistently addressed.

### 4.1 Self-management functions and active patient involvement

The mapped evidence indicates that current interventions mainly support self-management through education, oral self-care skills, symptom monitoring, and behavioral reinforcement. However, the intensity of self-management support varied substantially. Some interventions extended beyond information provision by incorporating daily self-assessment, individualized feedback, goal setting, reminders, or ongoing guidance. For example, the OrCaSS intervention incorporated self-assessment, selection of oral care strategies based on assessment results, jointly established self-management goals, and adherence reminders (13), whereas mobile- and telephone-based interventions supported symptom recording, tailored guidance, and communication with healthcare professionals (15, 17). These components represent a greater degree of active patient involvement than education alone.

Nevertheless, explicit goal setting, problem-solving, and psychosocial self-management were less consistently addressed across the included studies. This suggests that many existing interventions primarily support the performance of prescribed self-care behaviors rather than the broader capacity to recognize changing symptoms, make management decisions, and adapt self-management strategies over time. This pattern is noteworthy because established self-management frameworks emphasize problem-solving, decision-making, resource utilization, patient–professional partnership, and self-tailoring as core self-management skills (28). In cancer care specifically, McCorkle et al. emphasized that self-management should enable patients and families to participate actively in managing care across the cancer continuum rather than function solely as recipients of professional instruction (29). Taken together, these findings suggest that future oral mucositis interventions should move beyond knowledge transfer and prescribed oral-care behaviors toward more comprehensive support for patients’ self-management capabilities.

### 4.2 Heterogeneity across populations and treatment contexts

The substantial heterogeneity in populations and treatment contexts is also important when interpreting the mapped evidence. As identified in this review, most interventions were developed for patients receiving radiotherapy or chemoradiotherapy, particularly those with head and neck or nasopharyngeal cancer, whereas three studies involved hematopoietic stem cell transplantation, two focused on chemotherapy, and only one specifically addressed pediatric patients. These treatment contexts involve different self-management demands. External literature indicates that oral mucositis during head and neck radiotherapy typically develops progressively over the course of treatment, creating repeated opportunities for oral assessment, behavioral reinforcement, and stage-specific guidance (30). By contrast, patients undergoing hematopoietic stem cell transplantation face additional challenges related to intensive treatment, infection risk, nutritional compromise, and functional burden, which may require more comprehensive and closely integrated supportive care (31).

Within the included studies, Schmidt et al. implemented an interdisciplinary self-management program involving exercise therapists and nurses in patients undergoing hematopoietic stem cell transplantation, illustrating how self-management support in this setting may need to be embedded within multidisciplinary inpatient care (16). However, the study was single-center and nonrandomized, which limits the transferability of its findings to other treatment contexts. Population differences further constrain direct comparison. The only pediatric study, conducted by Bezerra et al. incorporated games, video demonstrations, positive reinforcement, and repeated oral assessment, highlighting the need for developmentally appropriate self-management support (21). Therefore, interventions developed for adults undergoing head and neck radiotherapy should not be assumed to be directly transferable to pediatric patients or those undergoing hematopoietic stem cell transplantation. Rather, self-management support should be interpreted and developed in relation to the specific treatment trajectory, care environment, and needs of the target population.

### 4.3 Continuity, personalization, and digital support

The continuity and personalization of self-management support also varied considerably. Most interventions remained embedded within hospital-based treatment, whereas only a limited number explicitly extended support beyond clinical encounters through telephone follow-up, mobile applications, WeChat-based continuity of care, or mobile diaries (15, 17, 23, 25). Such approaches may be particularly relevant for oral mucositis because symptoms and self-care needs continue between scheduled clinical visits and after patients return home. Some interventions further tailored support according to mucositis severity, self-care ability, treatment stage, or patient-reported problems (12, 15, 18–20, 24), although individualized adjustment was not consistently implemented across studies.

Digital approaches may provide an opportunity to strengthen this continuity. Five studies incorporated digital components, including mobile applications, WeChat, mobile diaries, video-based education, or ward-based educational media (15, 21, 23, 25, 27). However, these approaches were still largely used for information delivery, behavioral guidance, self-recording, or communication, and only some incorporated dynamic self-assessment or individualized feedback (15, 23, 25). The included studies also provided little information on patients’ digital access, eHealth literacy, or socioeconomic barriers to technology use. External literature suggests that eHealth literacy, access to digital devices, and usability are important factors influencing the uptake of digital interventions in cancer care (32, 33). In addition, international guidance has emphasized the value of patient-reported assessment in oral mucositis management (34), suggesting that future digital self-management support could move beyond information provision toward dynamic patient-reported monitoring and tailored feedback. At the same time, digital accessibility and equity should be considered to avoid assuming that technology-based support is equally available or usable for all patients.

### 4.4 Interpretation of reported outcomes and evidence limitations

Finally, the reported outcomes should be interpreted cautiously. Although favorable findings were reported in several studies, including improvements in oral mucositis-related outcomes, quality of life, or nutritional indicators (14, 15), null and mixed findings were also observed. For example, Schmidt et al. reported no between-group difference in the primary outcome of health-related quality of life (16), Leppla et al. reported only small effect sizes for several oral mucositis outcomes (13), and Bezerra et al. reported mixed findings regarding oral mucositis incidence and severity (21). Therefore, the available evidence should not be interpreted as establishing the overall effectiveness of self-management support interventions.

Nine of the 16 included studies used quasi-experimental designs, and several studies had relatively small samples or lacked a concurrent control group (12, 13, 17, 21). In addition, substantial heterogeneity existed in patient populations, treatment contexts, intervention components, and outcome measurement. Different studies used assessment systems such as the WHO oral mucositis grading scale, OAG, CTCAE, and RTOG criteria, together with various self-management, quality-of-life, nutritional, and investigator-developed measures, which limited direct comparison across studies. Because this scoping review did not undertake a formal critical appraisal or pool effect estimates, the current evidence is better interpreted as identifying potentially useful intervention components and contextual gaps than as demonstrating comparative or overall intervention effectiveness. Future research should use rigorous controlled designs, standardized outcome measures, and clearer intervention reporting to strengthen the evidence base.

## 5 Limitations

Several limitations should be considered when interpreting this review. First, the included studies were concentrated in a limited number of countries and regions, with a relatively high proportion conducted in China, which may limit the generalizability of the findings across different healthcare and cultural contexts. Second, although no language restrictions were applied during the search, potential language-access bias cannot be completely excluded. In addition, 32 reports could not be retrieved for full-text assessment, and four further reports contained insufficient full-text information for eligibility assessment, which may have introduced selection bias. Third, although we applied an operational definition of self-management support, considerable heterogeneity remained in intervention components, intensity, and delivery. Some studies also provided incomplete descriptions of intervention procedures, duration, or frequency, limiting comparison and replication. Fourth, outcome measures were heterogeneous, and several studies relied on investigator-developed questionnaires rather than standardized or validated instruments, which may reduce the comparability of findings across studies.

Finally, despite independent screening and data charting by two reviewers and pilot testing of the data-charting form, selection or charting errors cannot be completely excluded. Publication bias is also possible because studies reporting favorable findings may be more likely to be published. Moreover, no critical appraisal or risk-of-bias assessment was conducted and effect estimates were not pooled; therefore, the reported findings should not be interpreted as establishing the overall effectiveness of self-management support interventions.

## 6 Conclusion

Existing studies have reported potential improvements in oral mucositis-related clinical outcomes, self-management-related outcomes, quality of life, and nutritional status following self-management support interventions. However, the findings were heterogeneous and included favorable, null, and mixed results. Given the predominance of quasi-experimental evidence, variation in outcome measurement, and the absence of critical appraisal, the current evidence should not be interpreted as establishing overall intervention effectiveness. Future research should use theory-informed intervention designs, standardized outcome measures, and rigorous controlled studies to strengthen the evidence for self-management support in cancer treatment-related oral mucositis.

## Supplementary material

Supplementary File 1 Search strategies.

Supplementary File 2 Completed PRISMA-ScR checklist.
