# Supplementary material for: Self-management support interventions for patients with cancer treatment-related oral mucositis: a scoping review
Source: Front Oncol. 2026 Oct 2;16:1950737. doi: 10.3389/fonc.2026.1950737 (PMC13673534; doi:10.3389/fonc.2026.1950737)
Supplement: Supplementary file 1 [file SupplementaryFile2.pdf]

PRISMA-ScR Checklist

Preferred Reporting Items for Systematic reviews and Meta-Analyses extension for Scoping Reviews

Manuscript: "Self-management support interventions for patients with cancer treatment-related oral mucositis: A scoping review"

Note: Page numbers refer to Manuscript_clean(2).docx. The item structure follows PRISMA-ScR (Tricco et al., 2018). Where an item was not undertaken or is only partly reported, this is stated explicitly.

Section / topic	Item #	PRISMA-ScR reporting requirement	Reported on page(s) / location
**TITLE**			
Title	1	Identify the report as a scoping review.	p. 1
**ABSTRACT**			
Structured summary	2	Summarize the background/objective, eligibility and information sources, charting methods, results, and conclusions.	pp. 1–2
**INTRODUCTION**			
Rationale	3	Explain the rationale in relation to existing knowledge and why a scoping review approach is appropriate.	pp. 2–3 (topic rationale reported; explicit justification for the scoping-review approach is not separately stated)
Objectives	4	State the review objective(s) and/or questions, including the main population, concept, and context elements.	pp. 3–4
**METHODS**			
Protocol and registration	5	State whether a protocol exists, where it can be accessed, and any registration information.	p. 3 (OSF DOI provided)
Eligibility criteria	6	Specify eligibility characteristics, including population/concept/context, study types, language/date restrictions, and relevant rationale.	pp. 4–5
Information sources	7	List all information sources searched and report the date of the most recent search.	p. 5
Search	8	Provide a reproducible electronic search strategy for at least one database, including any limits.	p. 5; Supplementary File 1
Selection of sources of evidence	9	Describe the screening and eligibility process used to select sources of evidence.	pp. 5–6
Data charting process	10	Describe how the charting form was developed/tested, who charted the data, whether charting was independent/duplicate, and how disagreements were handled.	pp. 5–6
Data items	11	List the variables/data items sought and note any assumptions or simplifications used.	pp. 5–6; Table 1 (variables reported)
Critical appraisal of individual sources	12	If critical appraisal was conducted, explain why and describe the methods and use of appraisal results.	N/A – critical appraisal was not conducted; see p. 17
Synthesis of results	13	Describe how charted data were handled, categorized, summarized, and synthesized.	p. 6
**RESULTS**			
Selection of sources of	14	Report numbers identified, screened, assessed, excluded with reasons, and included, preferably using a flow diagram.	pp. 6–7; Figure 1

Section / topic	Item #	PRISMA-ScR reporting requirement	Reported on page(s) / location
evidence			
Characteristics of sources of evidence	15	Present the charted characteristics of each included source and provide citations.	pp. 7–10; Table 1
Critical appraisal within sources	16	If critical appraisal was conducted, present the appraisal results.	N/A – critical appraisal was not conducted
Results of individual sources	17	Present relevant charted data from individual included sources in relation to the review questions.	pp. 8–10; Table 1
Synthesis of results	18	Summarize the charted results in relation to the review questions and objectives.	pp. 9–12 (Sections 3.3.5 and 3.4)
**DISCUSSION**			
Summary of evidence	19	Summarize the main concepts, themes, patterns, and types of evidence and relate them to the review questions and relevant groups.	pp. 12–16 (Sections 4.1–4.4)
Limitations	20	Discuss limitations of the scoping review process and the included evidence where relevant.	pp. 16–17
Conclusions	21	Provide an overall interpretation linked to the review questions, with implications and/or next steps.	pp. 17–18
**FUNDING**			
Funding	22	Report funding for the scoping review and included sources, and describe the role of the review funder(s).	p. 18 (review funding source reported)

Reference: Tricco AC, Lillie E, Zarin W, et al. PRISMA Extension for Scoping Reviews (PRISMA-ScR): Checklist and Explanation. Ann Intern Med. 2018;169(7):467–473. doi:10.7326/M18-0850.
