# Supplementary material for: Self-management support interventions for patients with cancer treatment-related oral mucositis: a scoping review
Source: Front Oncol. 2026 Oct 2;16:1950737. doi: 10.3389/fonc.2026.1950737 (PMC13673534; doi:10.3389/fonc.2026.1950737)
Supplement: Supplementary file 2 [file SupplementaryFile1.docx]

**Supplemental file 1--- Database,the Search Strategy and Results**

| **Database** | **The search strategy** | **Results** |
| --- | --- | --- |
| **PubMed** | #1 (((((((((Cancer[Title/Abstract]) OR (Neoplasm*[Title/Abstract])) OR (tumor*[Title/Abstract])) OR (tumour*[Title/Abstract])) OR (Oncology[Title/Abstract])) OR (chemotherap*[Title/Abstract])) OR (radiotherap*[Title/Abstract])) OR (chemoradiotherap*[Title/Abstract])) OR ("hematopoietic stem cell transplantation"[Title/Abstract])) OR (HSCT[Title/Abstract]) | 4,372,977 |
|  | #2 "Neoplasms"[MeSH Terms] OR "Radiotherapy"[MeSH Terms] OR "Drug Therapy"[MeSH Terms] OR "Hematopoietic Stem Cell Transplantation"[MeSH Terms] | 5,505,456 |
|  | #3 #1 OR #2 | 6,876,974 |
|  | #4 ((("Oral Mucositis"[Title/Abstract]) OR (Stomatitis[Title/Abstract])) OR ("oral mucosal injur*"[Title/Abstract])) OR ("radiation stomatitis"[Title/Abstract]) | 21,999 |
|  | #5 "Mucositis"[MeSH Terms] OR "Stomatitis"[MeSH Terms] | 21,612 |
|  | #6 #4 OR #5 | 36,523 |
|  | #7 ((((((((((("self-management"[Title/Abstract]) OR (selfcare*[Title/Abstract])) OR ("self care"[Title/Abstract])) OR ("self-nursing"[Title/Abstract])) OR (education[Title/Abstract])) OR ("self-control"[Title/Abstract])) OR ("self-management behavior"[Title/Abstract])) OR ("mobile health"[Title/Abstract])) OR (mHealth[Title/Abstract])) OR ("nurse-led"[Title/Abstract])) OR (telehealth[Title/Abstract])) OR ("follow-up"[Title/Abstract]) OR (WeChat[Title/Abstract]) | 2,250,395 |
|  | #8 "Self Care"[MeSH Terms] OR "Health Education"[MeSH Terms] OR "Patient Education as Topic"[MeSH Terms] OR "Telemedicine"[MeSH Terms] OR "Mobile Applications"[MeSH Terms] OR "Follow-Up Studies"[MeSH Terms] OR "Nursing Care"[MeSH Terms] | 1,244,964 |
|  | #9 #7 OR #8 | 3,009,110 |
|  | #10 #3 AND #6 AND #9 | 1,578 |
| **Cochrane Library** | #1 (Cancer):ti,ab,kw OR (Neoplasm*):ti,ab,kw OR (tumor*):ti,ab,kw OR (tumour*):ti,ab,kw OR (Oncology):ti,ab,kw OR (chemotherap*):ti,ab,kw OR (radiotherap*):ti,ab,kw OR (chemoradiotherap*):ti,ab,kw OR ("hematopoietic stem cell transplantation"):ti,ab,kw OR (HSCT):ti,ab,kw | 316501 |
|  | #2 MeSH descriptor: [Neoplasms] explode all trees OR MeSH descriptor: [Radiotherapy] explode all trees OR MeSH descriptor: [Drug Therapy] explode all trees | 281923 |
|  | #3 #1 OR #2 | 468614 |
|  | #4 ("oral mucositis"):ti,ab,kw OR (stomatitis):ti,ab,kw OR ("oral mucosal injur*"):ti,ab,kw OR ("radiation stomatitis"):ti,ab,kw | 6694 |
|  | #5 MeSH descriptor: [Mucositis] explode all trees OR MeSH descriptor: [Stomatitis] explode all trees | 1887 |
|  | #6 #4 OR #5 | 6991 |
|  | #7 ("self-management"):ti,ab,kw OR (selfcare*):ti,ab,kw OR ("self care"):ti,ab,kw OR ("managing oneself"):ti,ab,kw OR ("self-nursing"):ti,ab,kw OR (education):ti,ab,kw OR ("self-control"):ti,ab,kw OR ("self-management behavior"):ti,ab,kw OR ("mobile health"):ti,ab,kw OR (mHealth):ti,ab,kw OR ("nurse-led"):ti,ab,kw OR (telehealth):ti,ab,kw OR ("follow-up"):ti,ab,kw OR (WeChat):ti,ab,kw | 495509 |
|  | #8 MeSH descriptor: [Self Care] explode all trees OR MeSH descriptor: [Health Education] explode all trees OR MeSH descriptor: [Patient Education as Topic] explode all trees OR MeSH descriptor: [Telemedicine] explode all trees OR MeSH descriptor: [Mobile Applications] explode all trees OR MeSH descriptor: [Follow-Up Studies] explode all trees OR MeSH descriptor: [Nursing Care] explode all trees | 98966 |
|  | #9 #7 OR #8 | 501124 |
|  | #10 #3 AND #6 AND #9 | 1107 |
| **Web of Science** | #1 ((((((((((TI=(Cancer)) OR TI=(Neoplasm*)) OR TI=(tumor*)) OR TI=(tumour*)) OR TI=(Oncology)) OR TI=(chemotherap*)) OR TI=(radiotherap*)) OR TI=(chemoradiotherap*)) OR TI=("hematopoietic stem cell transplantation")) OR TI=(HSCT)) OR ((((((((((AB=(Cancer)) OR AB=(Neoplasm*)) OR AB=(tumor*)) OR AB=(tumour*)) OR AB=(Oncology)) OR AB=(chemotherap*)) OR AB=(radiotherap*)) OR AB=(chemoradiotherap*)) OR AB=("hematopoietic stem cell transplantation")) OR AB=(HSCT)) | 3,268,410 |
|  | #2 (((TI=("oral mucositis")) OR TI=(stomatitis) OR TI=("oral mucosal injur*") OR TI=("radiation stomatitis")) OR ((AB=("oral mucositis")) OR AB=(stomatitis) OR AB=("oral mucosal injur*") OR AB=("radiation stomatitis"))) | 10,390 |
|  | #3 (((((((((((((TI=("self-management")) OR TI=(selfcare*)) OR TI=("self care")) OR TI=("managing oneself")) OR TI=("self-nursing")) OR TI=(education)) OR TI=("self-control")) OR TI=("self-management behavior")) OR TI=("mobile health")) OR TI=(mHealth)) OR TI=("nurse-led")) OR TI=(telehealth)) OR TI=("follow-up")) OR TI=(WeChat)) OR (((((((((((((AB=("self-management")) OR AB=(selfcare*)) OR AB=("self care")) OR AB=("managing oneself")) OR AB=("self-nursing")) OR AB=(education)) OR AB=("self-control")) OR AB=("self-management behavior")) OR AB=("mobile health")) OR AB=(mHealth)) OR AB=("nurse-led")) OR AB=(telehealth)) OR AB=("follow-up")) OR AB=(WeChat)) | 1,374,385 |
|  | #4 #1 AND #2 AND #3 | 442 |
| **CINAHL** | #1 TI Cancer OR TI Neoplasm* OR TI tumor* OR TI tumour* OR TI Oncology OR TI chemotherap* OR TI radiotherap* OR TI chemoradiotherap* OR TI "hematopoietic stem cell transplantation" OR TI HSCT OR AB Cancer OR AB Neoplasm* OR AB tumor* OR AB tumour* OR AB Oncology OR AB chemotherap* OR AB radiotherap* OR AB chemoradiotherap* OR AB "hematopoietic stem cell transplantation" OR AB HSCT | 757,951 |
|  | #2 MH "Neoplasms+" OR MH "Radiotherapy+" OR MH "Drug Therapy+" OR MH "Hematopoietic Stem Cell Transplantation+" | 854379 |
|  | #3 #1 OR #2 | 1128373 |
|  | #4 TI "oral mucositis" OR TI stomatitis OR TI "oral mucosal injur*" OR TI "radiation stomatitis" OR AB "oral mucositis" OR AB stomatitis OR AB "oral mucosal injur*" OR AB "radiation stomatitis" | 3570 |
|  | #5 MH "Mucositis+" OR MH "Stomatitis+" | 5645 |
|  | #6 #4 OR #5 | 7228 |
|  | #7 TI "self-management" OR TI selfcare* OR TI "self care" OR TI "managing oneself" OR TI "self-nursing" OR TI education OR TI "self-control" OR TI "self-management behavior" OR TI "mobile health" OR TI mHealth OR TI "nurse-led" OR TI telehealth OR TI "follow-up" OR TI WeChat OR AB "self-management" OR AB selfcare* OR AB "self care" OR AB "managing oneself" OR AB "self-nursing" OR AB education OR AB "self-control" OR AB "self-management behavior" OR AB "mobile health" OR AB mHealth OR AB "nurse-led" OR AB telehealth OR AB "follow-up" OR AB WeChat | 743,964 |
|  | #8 MH "Self Care+" OR MH "Health Education+" OR MH "Patient Education as Topic+" OR MH "Telehealth+" OR MH "Mobile Applications+" OR MH "Follow-Up Studies+" OR MH "Nursing Care+" | 667591 |
|  | #9 #7 OR #8 | 1277583 |
|  | #10 #3 AND #6 AND #9 | 660 |
| **Embase** | #1 (Cancer):ti,ab,kw OR (neoplasm*):ti,ab,kw OR (tumor*):ti,ab,kw OR (tumour*):ti,ab,kw OR (oncology):ti,ab,kw OR (chemotherap*):ti,ab,kw OR (radiotherap*):ti,ab,kw OR (chemoradiotherap*):ti,ab,kw OR ("hematopoietic stem cell transplantation"):ti,ab,kw OR (HSCT):ti,ab,kw | 6242259 |
|  | #2 'neoplasm'/exp OR 'radiotherapy'/exp OR 'drug therapy'/exp OR 'hematopoietic stem cell transplantation'/exp | 6245221 |
|  | #3 #1 OR #2 | 8315542 |
|  | #4 ('oral mucositis'):ti,ab,kw OR (stomatitis):ti,ab,kw OR ('oral mucosal injur*'):ti,ab,kw OR ('radiation stomatitis'):ti,ab,kw | 29982 |
|  | #5 'oral mucositis'/exp OR 'stomatitis'/exp | 27777 |
|  | #6 #4 OR #5 | 49736 |
|  | #7 ('self-management'):ti,ab,kw OR (selfcare*):ti,ab,kw OR ('self care'):ti,ab,kw OR ('managing oneself'):ti,ab,kw OR ('self-nursing'):ti,ab,kw OR (education):ti,ab,kw OR ('self-control'):ti,ab,kw OR ('self-management behavior'):ti,ab,kw OR ('mobile health'):ti,ab,kw OR (mHealth):ti,ab,kw OR ('nurse-led'):ti,ab,kw OR (telehealth):ti,ab,kw OR ('follow-up'):ti,ab,kw OR (WeChat):ti,ab,kw | 3496116 |
|  | #8 'self care'/exp OR 'self management'/exp OR 'health education'/exp OR 'patient education'/exp OR 'telemedicine'/exp OR 'mobile application'/exp OR 'follow up'/exp OR 'nursing care'/exp | 3351211 |
|  | #9 #7 OR #8 | 4,706616 |
|  | #10 #3 AND #6 AND #9 | 2684 |
| **中国知网（CNKI）** | #1 TKA = ('肿瘤' + '癌症' + '癌' + '放疗' + '化疗' + '放化疗' + '造血干细胞移植') | 1967800 |
|  | #2 TKA = ('口腔黏膜炎' + '口腔炎' + '放射性口腔炎') | 11400 |
|  | #3 TKA = ('自我管理' + '自我照护' + '自我控制' + '自我护理' + '健康教育' + '自我管理行为' + '随访' + '移动健康' + '微信') | 1026500 |
|  | #4 #1 * #2 * #3【TKA=('肿瘤'+'癌症'+'癌'+'放疗'+'化疗'+'放化疗'+'造血干细胞移植')*('口腔黏膜炎'+'口腔炎'+'放射性口腔炎')*('自我管理'+'自我照护'+'自我控制'+'自我护理'+'健康教育'+'自我管理行为'+'随访'+'移动健康'+'微信')】 | 141 |
| **万方数据库** | #1 主题:("肿瘤" or "癌症" or "癌" or "放疗" or "化疗" or "放化疗" or "造血干细胞移植") | 5,146,589 |
|  | #2 主题:("口腔黏膜炎" or "口腔炎" or "放射性口腔炎") | 10,511 |
|  | #3 主题:("自我管理" or "自我照护" or "自我控制" or "自我护理" or "健康教育" or "自我管理行为" or "随访" or "移动健康" or "微信") | 1,382,133 |
|  | #4 #1 AND #2 AND #3  【主题:(("肿瘤" or "癌症" or "癌" or "放疗" or "化疗" or "放化疗" or "造血干细胞移植")) and 主题:(("口腔黏膜炎" or "口腔炎" or "放射性口腔炎")) and 主题:(("自我管理" or "自我照护" or "自我控制" or "自我护理" or "健康教育" or "自我管理行为" or "随访" or "移动健康" or "微信"))】 | 520 |
